# YOUPI: Your powerful and intelligent tool for segmenting cells from imaging mass cytometry data

**DOI:** 10.3389/fimmu.2023.1072118

**Published:** 2023-03-02

**Authors:** Yvonne Scuiller, Patrice Hemon, Marion Le Rochais, Jacques-Olivier Pers, Christophe Jamin, Nathan Foulquier

**Affiliations:** ^1^ LBAI, UMR 1227, Univ Brest, Inserm, Brest, France; ^2^ CHU de Brest, Brest, France

**Keywords:** imaging mass cytometry, cell segmentation, images, U-NET, new algorithm

## Abstract

The recent emergence of imaging mass cytometry technology has led to the generation of an increasing amount of high-dimensional data and, with it, the need for suitable performant bioinformatics tools dedicated to specific multiparametric studies. The first and most important step in treating the acquired images is the ability to perform highly efficient cell segmentation for subsequent analyses. In this context, we developed YOUPI (Your Powerful and Intelligent tool) software. It combines advanced segmentation techniques based on deep learning algorithms with a friendly graphical user interface for non-bioinformatics users. In this article, we present the segmentation algorithm developed for YOUPI. We have set a benchmark with mathematics-based segmentation approaches to estimate its robustness in segmenting different tissue biopsies.

## 1 Introduction

Immunohistochemistry and immunofluorescence are currently the most commonly used approaches for analyzing tissue biopsies. These techniques enable the use of four to six fluorescence-conjugated antibodies for detecting markers expressed in the same tissue, leading to a limited number of multiple staining combinations due to the low number of revelation channels that can be used together. Moreover, the properties of fluorescence-associated antibodies can overlap. Their overlapping complicates the process of using a complex fluorescence-conjugated antibody combination for later analysis in terms of diagnosis, prognosis, or treatment elaboration, thus leading to using only a small number of fluorochromes with non-overlapping signals (1). However, these challenges have recently been overcome by evolving approaches, such as the recent fluorescence-associated approach CO-Detection by indEXing (CODEX) (2). Additionally, new approaches based on the use of metal-tagged antibodies have resulted in the development of technologies such as Multiplexed Ion Beam Imaging Technology (MIBI) (3) and Imaging Mass Cytometry (IMC) (4). They ensure the detection of cellular marker expressions that do not interfere with each other. These techniques are significant for evaluating up to 40 markers on each cell simultaneously (5) and for preserving high-resolution spatial information (6).

The cell segmentation process extracts image information from the generated data, allowing for associating each detected marker and spatial coordinates with each cell (7). This step is essential for subsequent good-quality downstream analysis, such as in-depth immunophenotyping, which provides a powerful toolkit for understanding physiological processes and diagnostic methods.

Segmentation consists of annotating the image to assign specific pixels to objects and thus gather pixels with already known criteria. When applied to biological data, segmentation is critical for working at a single-cell level. Thus, cell segmentation remains a complex exercise, primarily because of the cells’ irregular shapes, heterogeneous density, and unevenly distributed membrane marking (8). Few attempts have been made to handle the task with simple mathematical approaches, such as CellProfiler, which has been developed using threshold pixel intensity, the watershed method, or the propagation algorithm (9). Pipelines based on the combination of Ilastik (10) and CellProfiler softwares (11) and, more recently, QuPath software (12) are some commonly used tools in research laboratories for the cell segmentation of IMC data. However, these solutions are time consuming, false-positive detections are still frequently observed, and they are not available as easy-to-use kits for non-computer users. The need for robust, accurate segmentation techniques with quick, easy access remains challenging.

Meanwhile, artificial intelligence is beginning to play an important role in scientific research, mainly through machine learning approaches. Techniques such as clustering, decision trees, and deep neural networks are already being used to segment magnetic resonance imaging (MRI) data (13). Machine learning is used to analyze and learn common characteristics from available data. Although cell segmentation remains a challenge even with machine learning, it is useful when dealing with the heterogeneity of cell shapes from different tissues (14).

For decades, machine learning has been used for image analysis in the biomedical field for classification tasks (15). In our case, segmentation is a particular type of feature extraction. Due to recent advances in machine learning, we can now choose from a variety of network architectures, such as GAN (16), TRANSFORMER (17), and U-Net (18), according to the task required. If sufficient and varied data are provided, this type of network can capture the heterogeneity of cell shapes.

Here, we present YOUPI (an acronym for YOUr Powerful and Intelligent tool), an innovative tool for cell segmentation in tissue whose images are generated by an IMC. YOUPI works with a U-shaped neural network (19–21). Better known as “U-Net” (18), this type of network is adapted to answer the question of cell segmentation with biological data and is fascinating since it requires only a small amount of data to be trained. Therefore, the U-Net appears suitable for analyzing rare precious tissue samples. We developed YOUPI to provide a tool with a friendly graphical user interface for obtaining cell segmentation masks.

## 2 Materials and methods

### 2.1 Antibody conjugation

Carrier-free antibodies were conjugated to metal tags using the MaxPar^®^ labeling kit (Fluidigm) following the manufacturer’s instructions. Antibodies were stored at 500 μg/ml in a stabilizing solution (Candor Biosciences) at 4°C.

### 2.2 Tissue staining and IMC image acquisition

All samples issued from different patients were included in a registered autoimmune disease or tumor tissue collection, and the present study was conducted following national and institutional guidelines in compliance with the Helsinki Declaration and after approval by our institutional review board.

Formalin-Fixed and paraffin-embedding (FFPE) sections of 4 μm thickness from the salivary glands of patients with Sjögren’s syndrome, intestinal cancer, small cell lung cancer, and non-small cell lung cancer were cut and placed onto glass slides. Sections were de-paraffinized with xylene and carried through sequential rehydration from 100% Ethanol to 70% Ethanol before being transferred to a Tris buffer solution (TBS). Heat-induced antigen retrieval was performed in a water bath at 95° C for 30 min in Tris/EDTA buffer (10mM Tris, 1mM EDTA, pH9). Slides were cooled to room temperature (RT) and subsequently blocked using phosphate-buffered saline (PBS) with 3% BSA for 30 min at RT. Each slide was incubated with 100 μl of the metal-tagged antibody cocktail (**Table 1**) overnight at 4° C. Then, the slides were washed three times with PBS and labeled with a 1:500 dilution of Intercalator-Ir (Fluidigm) in TBS for 2 min at RT. Slides were briefly washed with H2O and air dried before IMC acquisition. Data were acquired on a Hyperion Imaging System™ coupled to a Helios Mass Cytometer (Fluidigm) at a laser frequency of 200 Hz and a laser power of 3 dB. For each recorded region of interest (ROI), stacks of 16-bit single-channel TIFF files were exported from MCD binary files using MCD™ Viewer 1.0 software (Fluidigm).

**Table 1:** Panels of markers for the imaging mass cytometry acquisition.

Salivary gland description	Selected for segmentation	Lung description	Selected for segmentation	Intestine description	Selected for segmentation	Kidney description	Selected for segmentation
CD38		CD38		CD83		CD38	
CD204		CD21		CD38		Ki-67	
Vimentin		Vimentin		CD21		Vimentin	**X**
CD14		CD14		CD23		CD14	
T-bet		T-bet		DC- LAMP		T-bet	
CD34		CD34	**X**	T-bet		CD16	
CD163		CD163		CD34		Collagen I	
PanKeratin	**X**	PanKeratin	**X**	CD163		CD45RA	**X**
CD11b		GATA3		PanKeratin	**X**	TNFalpha	
TSLP		CD274		GATA-3		IL-1beta	
CD31		CD31	**X**	CD274		CD31	
Ki-67		Ki-67		CD31		CD45	**X**
IgD		CD223		Ki-67		CD197	
IgM		TIM3		IgD		IL-17	
FoxP3		FoxP3		AID		FoxP3	
CD4		CD86		FoxP3		CD4	
CD117		CD117		CD86		CD69	
CD68	**X**	CD68	**X**	CD68	**X**	CD68	**X**
Bcl6		CD152		Bcl6		IL-6	
CD20	**X**	CD20	**X**	CD20	**X**	CD20	**X**
CD8a	**X**	CD8a	**X**	CD8a	**X**	CD8a	**X**
CD138	**X**	CD138	**X**	CD138	**X**	CD138	**X**
MPO	**X**	MPO	**X**	MPO	**X**	MPO	**X**
Flt3 ligand		CD279		CD279		Catenin	
CD56		CD56	**X**	CD56		CD56	
CD106		Granzyme B		Granzyme B		Granzyme B	
CD127		CD196		CD185		CD127	**X**
Collagen I		Collagen I		CD3	**X**	CD185	
CD3	**X**	CD3	**X**	CD27		CD3	
CD27		CD27		Caspase-3		C5b9	
Caspase-3		Caspase-3		Podoplanin	**X**	Caspase 3	
Podoplanin	**X**	Podoplanin	**X**	HLA-DR		IFNgamma	
HLA-DR		HLA-DR	**X**	CD4		HLA-DR	**X**
pS6		CD4	**X**	IgM		WT1	
CD135		IgM		CD11c		Collagen IV	
CXCL13		SMA		CD103		PanKeratin	
IgA		IgA		STAT3		CD15	
IgG		IgG		IgG		CD279	
CD183		CXCL13		DNA	**X**	SMA	**X**
CD11c		CD11c	**X**			IgG	
DNA	**X**	DNA	**X**			DNA	**X**

To prepare for training the neural network, cell-based morphological segmentation was carried out using supervised pixel classification with the Ilastik toolkit (22) to identify nuclei, membranes, and backgrounds. CellProfiler software (11) was used to segment the resulting probability maps. Inputs of 16-bit TIFF images with their corresponding segmentation masks were uploaded to histoCAT analysis toolbox (23) to open a session data analysis. Dimensionality reduction and unsupervised FLOWSOM clustering for 16-bit single images were performed using Cytobank on FCS files.

### 2.3 Workstation used for training

The model was trained for about 30 minutes on a workstation with 125 GB of RAM and an Intel Core i9-10920X CPU.

### 2.4 IMC training dataset pre-processing

The dataset used to train the neural network consisted of 81 patches randomly selected, including 30 patches from salivary glands of a patient with Sjögren’s syndrome, 20 patches from an intestine, five patches from a small cell lung cancer, and 26 patches from a non-small cell lung cancer. The heterogeneity of the tissues was expected to improve the capacity for detecting cells of various shapes. The trained model was evaluated on 90 new patches from salivary glands.

Cell-based morphological segmentation was conducted using supervised pixel classification by Ilastik (22) to identify nuclei, membranes, and backgrounds, followed by CellProfiler analysis (11) to segment the resulting probability maps. Masks obtained from this segmentation were manually corrected, based on the co-detection of the nuclei signals and membrane signals, and used as the ground truth for the network training.

### 2.5 Training and characteristics of the neural network

YOUPI is based on U-Net architecture. It consists of convolutional neural networks (CNN) arranged to perform semantic segmentation and contains two parts. The first contracting one has a typical CNN architecture. Each block of this path consists of two 3×3 convolution layers in a row, followed by a rectified linear unit (ReLU) and a 2×2 max-pooling operation. This procedure is performed four times. The second expansive path involves an oversampling of the feature map, followed by 2×2 convolutions at each stage. To enable precise localization, the feature map from the corresponding layer in the contracting path is cropped and concatenated onto the upsampled feature map. Two successive 3×3 convolutions follow this step to end on a ReLU. A 1x1 convolution is used for the final layer to reduce the feature map to the desired number of output channels (18).

The output is in the form of an image whose pixels have values between 0 and 1. To obtain a binary image, all pixels below 0.5 must be 0 for black, and all pixels greater than 0.5 must be 1 for white. The white part represents the cell, and the black part represents the background.

For the training, we used a binary cross-entropy loss function defined as follows:


, $$loss\left(y,ŷ\right)\;=-\frac{1}{N}\sum_{i=0}^{N}\left(y_{i}\;*\;log\left({ŷ}_{i}\right)+\left(1-y_{i}\right)*log\left(1-y_{i}\right)\right)$$

where N stands for the total number of pixels in an image, *y*_*i*_ represents the corresponding target value, and *ŷ*_*i*_ is for the predicted pixel probability. The cross-entropy loss compares the predicted probabilities with the ground truth values, and the loss is minimized during the training process. The network runs with the Adam’s optimizer, a stochastic gradient descent method, a batch size of 16, and 500 epochs with an early stop, using Keras/Tensorflow packages in Python 3.

To evaluate the performance of the YOUPI tool, we checked the value of the Intersection over Union (IoU) metric, also known as the Jaccard index.


$$Jaccard\left(A,B\right)=\frac{\left|\;A\;\cap^{}\;B\right|}{\left|A\;\cup^{}\;B\right|}$$

where A is the predictive mask, and B is the ground truth.

The IoU is one of the most frequently used metrics for evaluating a model of image segmentation (24). Mathematically, it represents the proportion of the area overlapping the target mask and the prediction output. Its value can vary between 0 and 1. The mean IoU of a patch is computed as the mean of the IoU of each cell in the patch.

### 2.6 YOUPI features

#### 2.6.1 Cell segmentation mask generation

For each ROI, stacks of 16-bit single-channel TIFF files were exported from MCD files using MCD™ Viewer 1.0 software (Fluidigm).

A first cell-based morphological segmentation was conducted, as described in section 2.4.

A second cell segmentation method was performed using the OME-TIFF files of the markers stacked in a single TIFF file with ImageJ software (v.1.8.0_172). The TIFF file was opened with QuPath software (v.0.3.2), and the image type was set to fluorescence. The segmentation process was then run with the optimal parameters for each ROI (filter function, signal intensity threshold, etc.), based on the iridium channel.

#### 2.6.2 FCS file exportation

Session data analysis was opened with 16-bit TIFF images. Their corresponding segmentation mask previously generated were uploaded in histoCAT (23) to export data in FCS files.

### 2.7 Statistical analysis

To extract quantitative data, FCS files were uploaded with OMIQ software. Data were expressed as mean ± SEMs. Statistical analyses were performed with GraphPad Prism (GraphPad Software, La Jolla, CA) using the Wilcoxon test for comparing the paired values. Significant differences were estimated at p< 0.05.

## 3 Results

### 3.1 Elaboration of the dataset for pre-processing the neural network

#### 3.1.1 Elaboration of the preliminary segmentation mask

**Figure 1:**
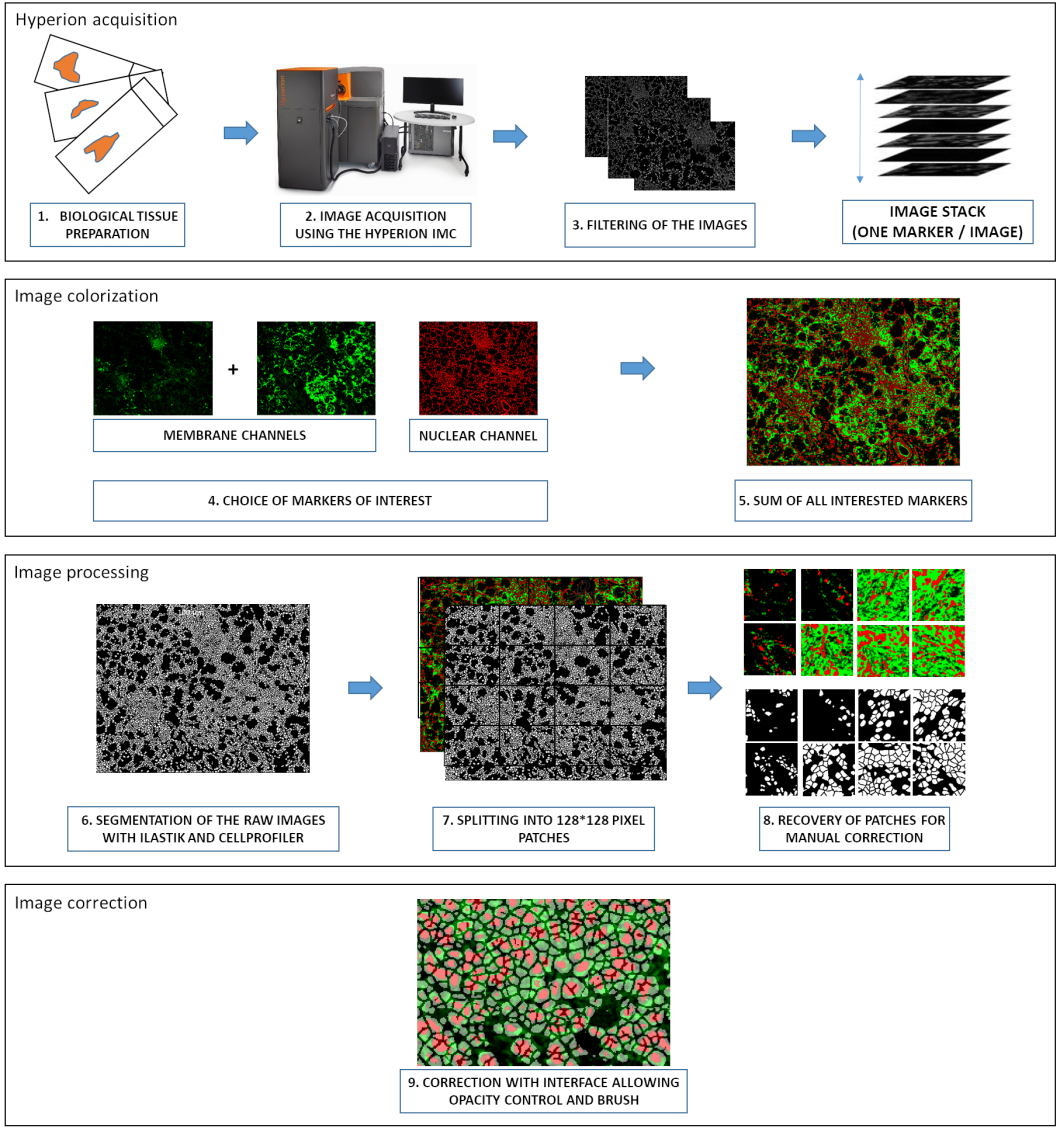
Graphical overview of the pre-processing steps to build the IMC training dataset. The pre-processing steps required to train the neural network of the YOUPI software consist of the following: 1. The preparation of biological tissues, 2. The acquisition of images with the HYPERION IMC, 3. The filtering of the images, 4. The selection of interesting markers, 5. The summation of interested markers, 6. The segmentation of raw images with Ilastik and CellProfiler, 7. The splitting of segmented images in 128×128 pixel patches, 8. The recovery of patches for manual correction, 9. The correction with the interface, allowing for opacity control and brush. IMC: Imaging Mass Cytometer.

The training dataset is the result of several steps. Once the preparation of the tissue slides is ready, IMC acquisition is performed (**Figure 1**). The acquisition produces a stack of images representing the same ROI with a different marker detected for each image. The MCD acquisition files from the Hyperion Imaging System™ are converted into TIFF format using an IMC file conversion tool (https://github.com/BodenmillerGroup/imctools). The marker panel was previously designed according to the tissue from which the IMC images were obtained. The interesting membrane markers are selected (**Table 1**; **Supplementary Figure 1**) and summed. In addition, image with stained nuclei is also selected. A graphical interface allows for colorizing the summed membrane markers in green and the nuclei marker in red before summing the two colors. Ilastik and CellProfiler software are then used to obtain a preliminary segmentation mask. Once generated, the binary mask of segmentation is split into patches of 128x128 pixels before manual correction.

#### 3.1.2 Segmentation from manual correction

**Figure 2:**
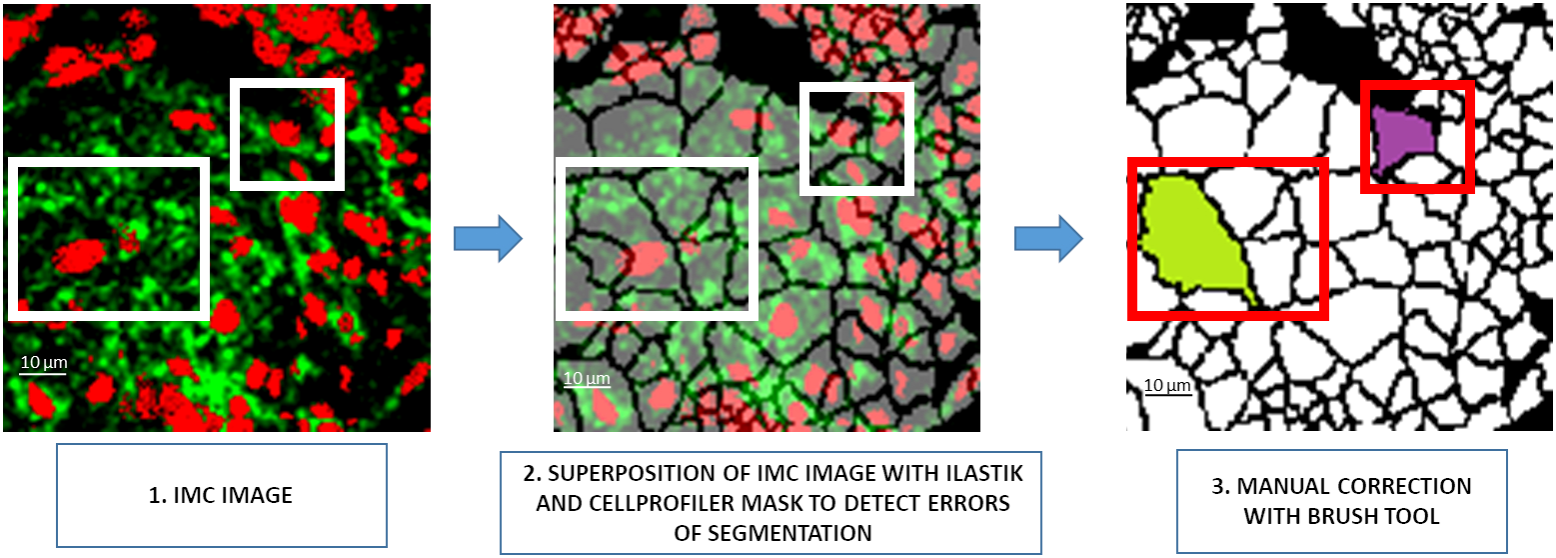
Visual interface preview for manual segmentation correction. 1.** **A 128×128 pixel patch of the IMC image, 2. Visualization of the segmentation mask generated by the Ilastik/CellProfiler pipeline to detect segmentation errors through the opacity management of the patch, 3. Manual correction of the segmentation error. IMC, Imaging Mass Cytometer.

Although time consuming and tedious, manual correction remains optimal for achieving accurate results in cell segmentation. To simplify the manual correction, a first tool was developed to superpose the segmentation mask generated by Ilastik and CellProfiler softwares and the patches of the IMC images. The control of opacity improves the visibility of the cell borders (**Figure 2**). A second tool was developed that provides a black-and-white brush to manually correct segmentation errors. In the example shown in **Figure 2**, the Ilastik and CellProfiler softwares segmented two cells instead of one in the largest rectangle and three cells instead of one in the smallest rectangle. A manual correction was thus necessary to obtain a single cell for both rectangles. The 81 patches used to train the neural network were generated accordingly.

### 3.2 YOUPI development

#### 3.2.1 Elaboration of the graphical interface

**Figure 3:**
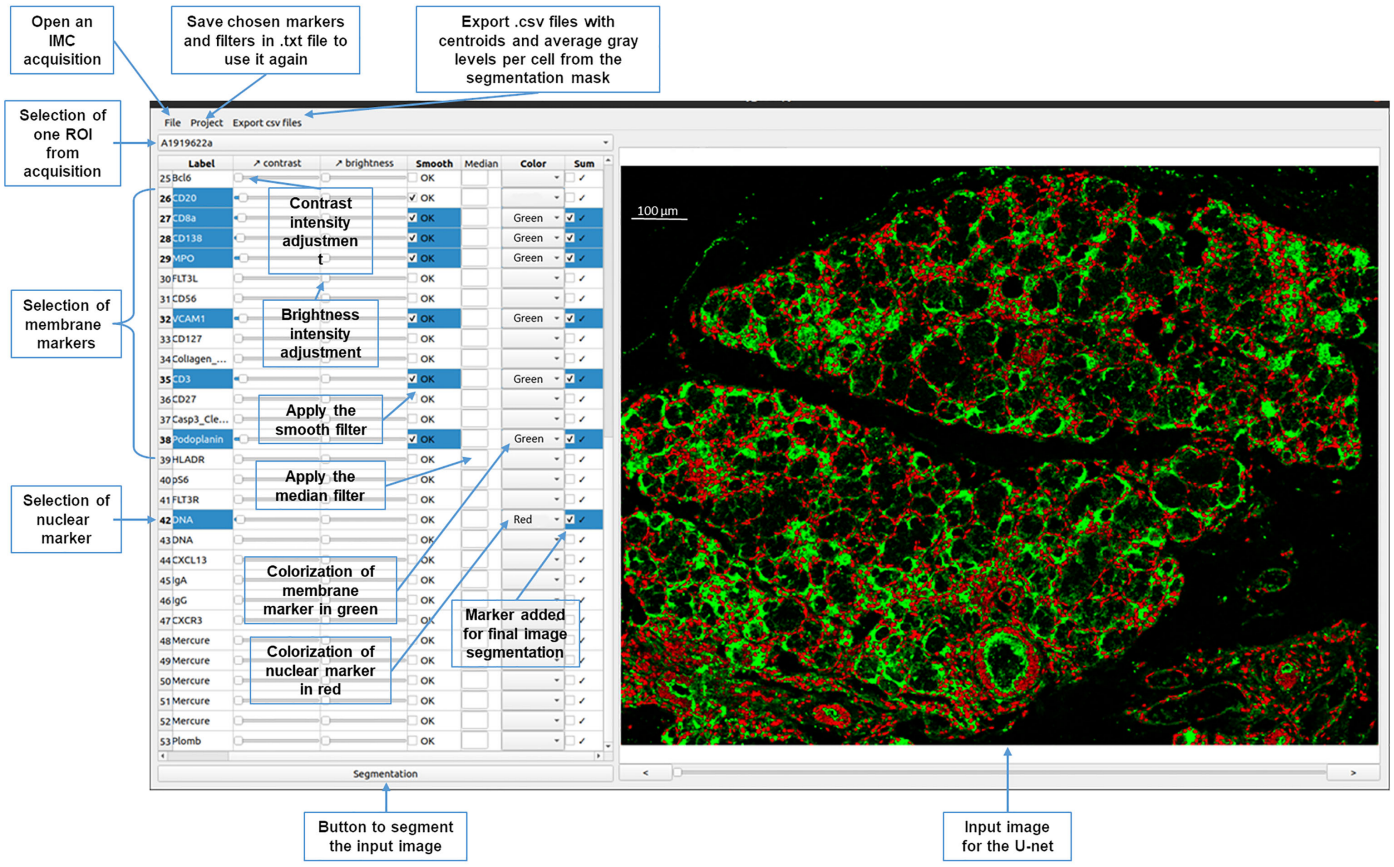
Graphical interface of the YOUPI software. An overview of the graphical interface and the ease-of-use functions of the YOUPI software.

To improve the usability of the U-Net segmentation, a graphical interface was developed to select the IMC images among the stack (**Figure 3**). The user can choose markers from the panel to generate an image that displays membrane (in green) and nuclei (in red) to provide input for the neural network. Median and smooth filters are used to enhance the quality of the image and the contrast and brightness adjusted for each chosen marker. Depending on the intensity of the signals, adjustment x2 to x3 is used for nuclei signal, and up to x10 for membrane signals. This adjustment process is essential for ensuring accurate cell segmentation.

#### 3.2.2 Input and output of the U-Net

**Figure 4:**
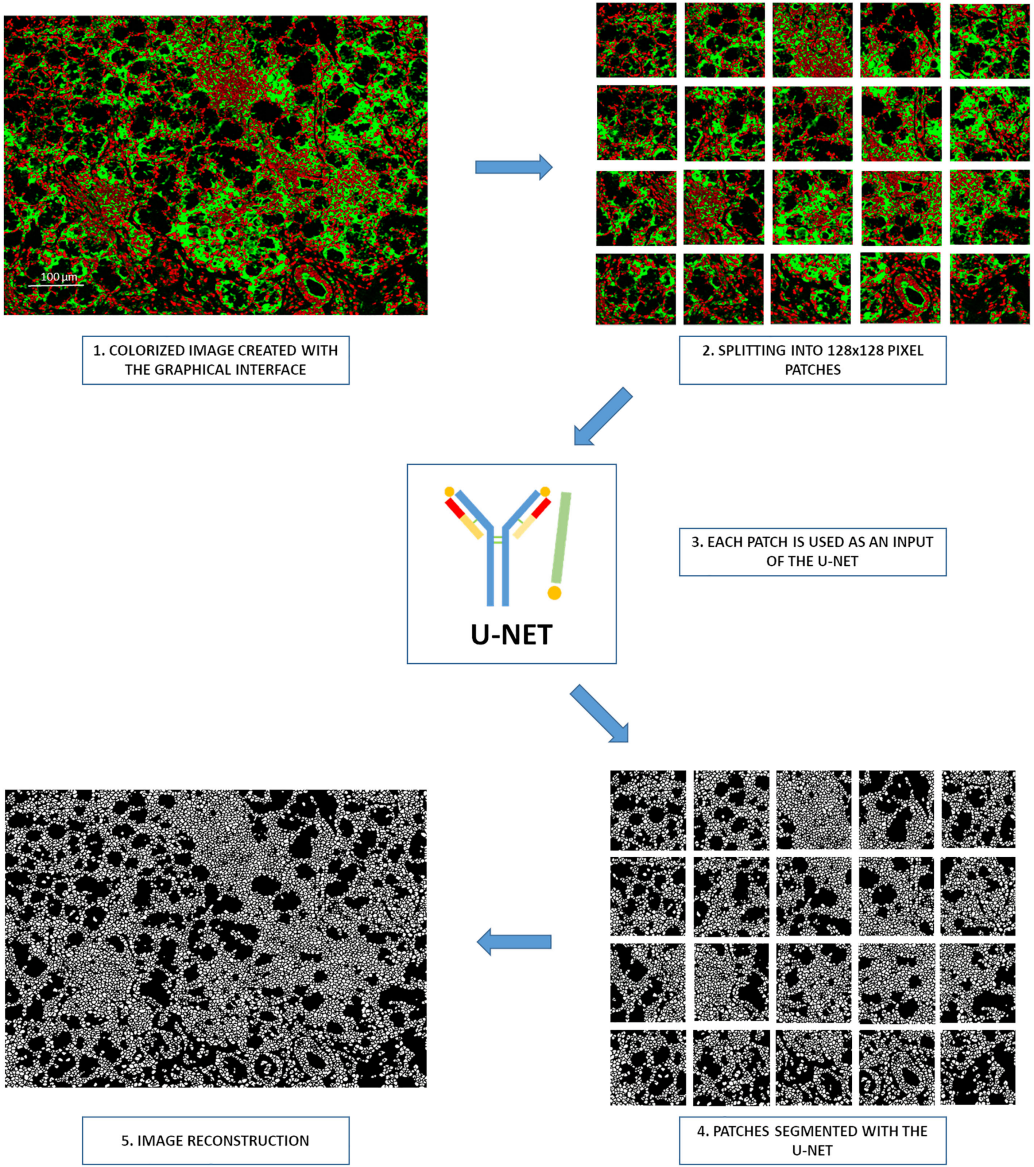
Processing steps for the Segmentation button of the graphical interface. 1. Creation of a colorized image by the graphical interface, 2. Splitting of images in 128×128 pixel patches, 3. Each patch serves as an input of the neural network, 4. Segmentation of all patches with the YOUPI tool, 5. Reconstruction of the entire image with the segmented patches.

The graphical interface allows users to build a colorized green-and-red image with the appropriate membrane and nuclei markers. When the image is considered sufficiently sharp, a simple click on the “Segmentation” button in the graphical interface is required. Once this button is pressed, different steps occur. The image is split into patches of 128×128 pixels. These patches are processed by the U-Net model to segment and generate binary cell segmentation masks. Once performed, the patches are gathered to rebuild the original image (**Figure 4**).

#### 3.2.3 Post-processing steps

**Figure 5:**
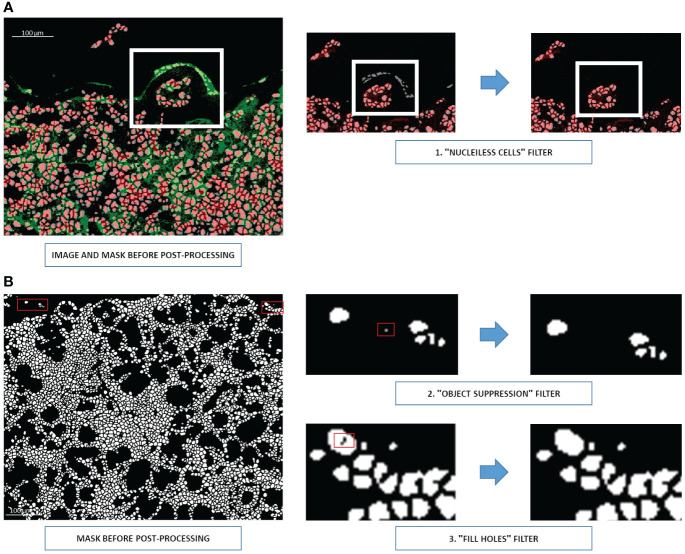
Three post-processing steps for U-Net output **(A)** Elimination of cells without nuclei signals (step 1). Nuclei are shown in red and membranes in green. A white rectangle indicates the region requiring corrections. **(B)** Removing one- or two-pixel objects (step 2) and filling holed nuclei (step 3). Cells are shown in white, and the background is shown in black. Red rectangles indicate the pixels requiring correction.

Once segmented, the image is ready for the post-processing steps. First, all cells in the mask whose coordinates do not show nuclei signals on the corresponding Hyperion acquisition are eliminated (**Figure 5A**). Second, all objects measuring only 1 or 2 pixels are removed, and the filter “fill holes” is used to fill abnormally holed nuclei (**Figure 5B**).

Overall, when users click on the “Segmentation” button from the graphical interface, the image that appears has gone through all the post-processing steps.

#### 3.2.4 CSV file generation for downstream analysis

Two types of CSV files can be generated from the post-processed segmented image. Since cells are defined as a list of identified pixels, the centroid of each cell is accessible, and the mean and median intensity of all markers for each cell can be calculated according to the pixel intensities in the raw IMC image.

**Figure 6:**
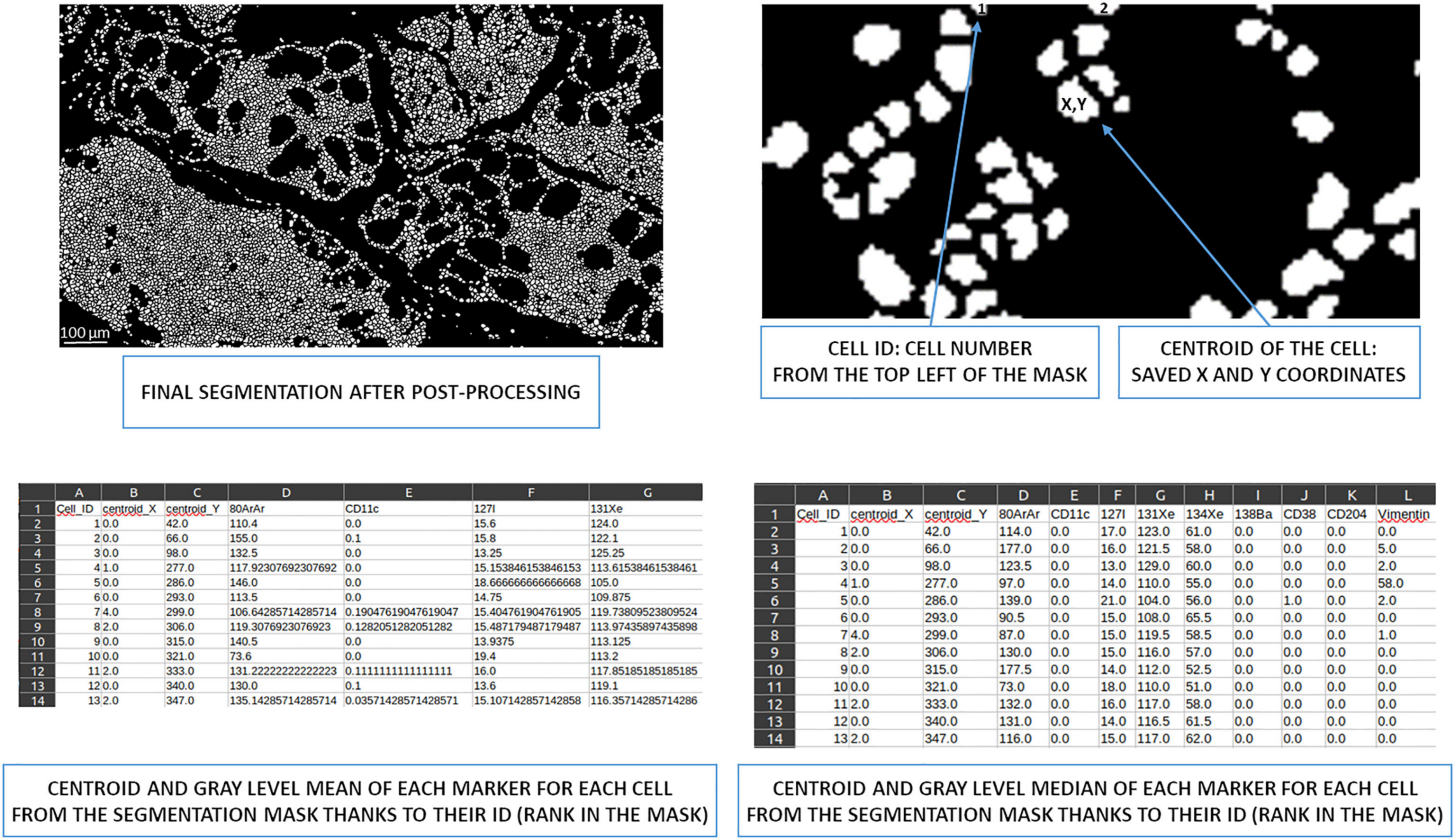
Overview of cell ID to obtain centroids and the mean/median of gray levels per marker. Based on the image from the post-processing segmentation, all cells were identified and had X and Y coordinates thanks to their centroid. This information is written in a CSV file. This file also contains the mean intensity of each marker for each cell. A second CSV file contains the median intensity of each marker for each cell. ID, identity.

Therefore, one CSV file (**Supplementary Table 1**) contains the centroid of each cell from the segmentation mask, followed by the mean intensity of all associated markers. A second CSV file (**Supplementary Table 2**) contains the centroid of each cell and, subsequently, the median intensity of all associated markers (**Figure 6**). The median intensity allows to ignore the aberrant high-intensity pixels that are frequently identified in IMC images. These CSV files can then be used for downstream supervised or unsupervised analysis.

### 3.3 Metrics for evaluating the YOUPI tool

To evaluate the performance of the YOUPI tool, we checked the value of the Intersection over Union (IoU) metric, also known as the Jaccard index. The IoU is one of the most frequently used metrics for evaluating a model of image segmentation (24). Mathematically, it represents the proportion of area overlapping between the target mask and the prediction output. Its value can vary between 0 and 1. The mean IoU of a patch is computed as the mean of the IoU of each cell in the patch.

After training, the U-Net reaches a mean IoU of 0.79 on 90 new patches. This value indicates that the results of the segmentation of cells by the U-Net are robust. Moreover, an image from kidney has been segmented without preliminary training of the U-Net with kidney tissues. The **Supplementary Figure 2** shows the efficient segmentation achieved using the YOUPI software, demonstrating its ability to segment multiple tissue types even without a training phase.

**Figure 7:**
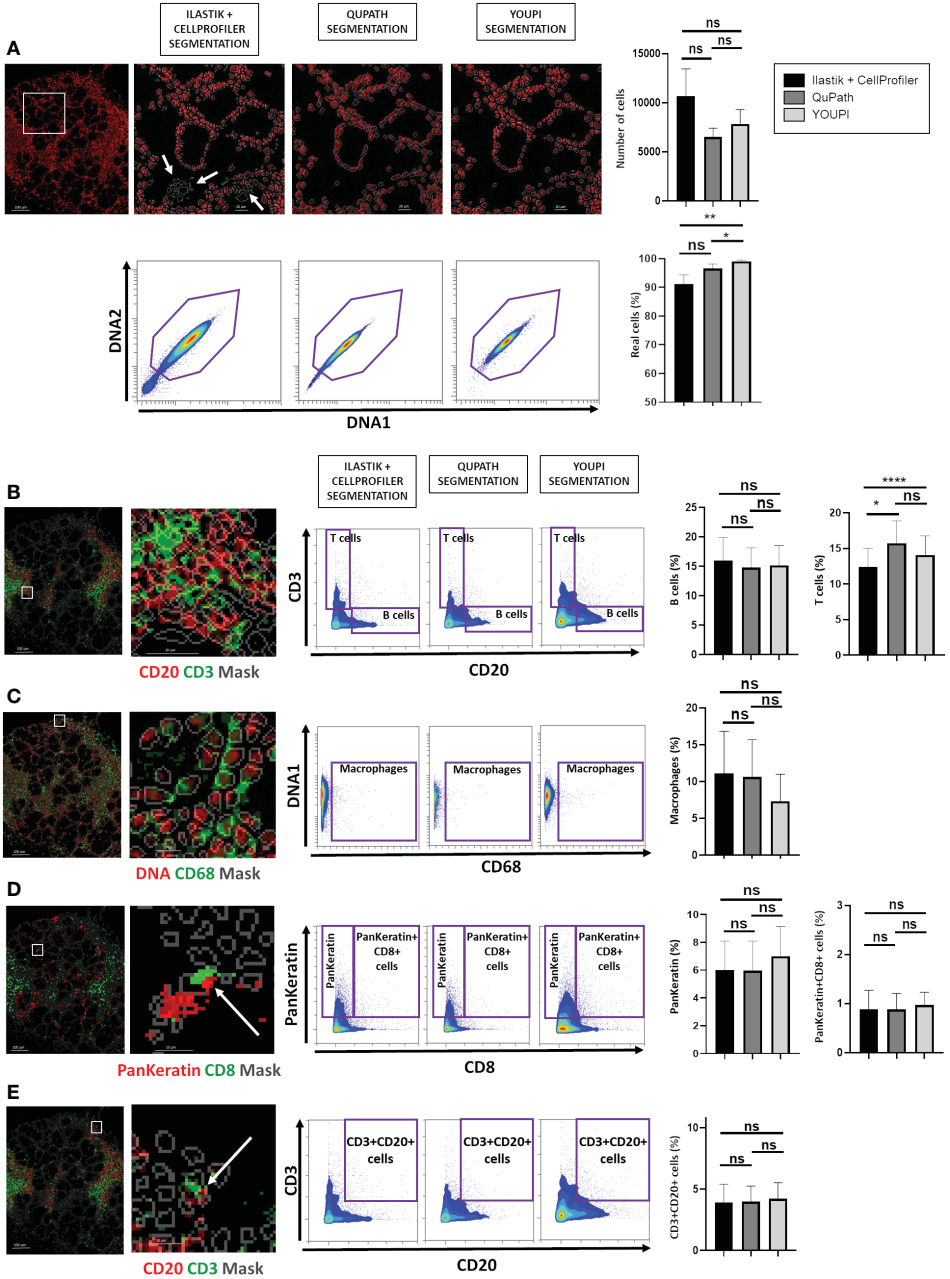
Biological metrics for evaluating cell segmentation performance. Eighteen ROI were segmented with Ilastik and CellProfiler, QuPath and YOUPI software. **(A)** Example of false cells (white arrows) found without DNA detection of the nuclei shown in red and the segmented mask of cells in gray. Two nuclei signals were used to establish the presence of existent cells. The number of cells and the percentage of real cells in the 18 ROI were determined. **(B)** Example of CD20 (red) and CD3 (green) detection with the YOUPI-generated mask shown in gray. The percentages of CD20+ B cells and of CD3+ T cells among the 18 ROIs were calculated. **(C)** Example of DNA1 (red) and CD68 (green) detection with the YOUPI-generated mask shown in gray. The percentage of CD68+ macrophages among the 18 ROIs was calculated. **(D)** Example of PanKeratin (red) and CD8 (green) detection, with the YOUPI-generated mask in gray. The white arrow indicates the detection of double-positive cells (white square). The percentages of PanKeratin+ cells and of double PanKeratin+ and CD8+ cells among the 18 ROIs were calculated. **(E)** Example of CD20 (red) and CD3 (green) detection with the YOUPI-generated mask shown in gray. The white arrow indicates the detection of double-positive cells (white square). The percentage of double CD3+ and CD20+ cells among the 18 ROIs was calculated. *p< 0.05, **p< 0.01, ****p< 0.001, ns, non-significant.

To further global quality, we sought an additional metric. We developed biological metrics, including the number of cells, the percentage of real cells, and the rate of false-positive cells. Real cells are objects characterized by at least a tenth percentile of the grayscale nuclei signal, below they are considered as non-existent cells, while false-positive cells correspond to objects co-expressing exclusive markers. Eighteen new ROIs from different tissues not used in the training data set and from different batches of data (two ROIs of one batch and two of another batch from salivary glands of patients with Sjögren’s syndrome, six from lung cancer, and eight from intestinal cancer) were segmented with the combination of Ilastik and CellProfiler, with QuPath and with YOUPI software, and the results were analyzed. Though not significantly different, the highest number of cells was found with the combination of Ilastik and CellProfiler software but was associated with the segmentation of non-existent cells (**Figure 7A**) and with the significantly lowest rate of real cells (91.2 ± 13.5%) compared with those detected with the YOUPI software (99.0 ± 2.1%, p< 0.01). There was no significant difference between the number of cells detected with the QuPath software and the YOUPI software. However, there were also significantly fewer real cells with the QuPath software (96.6 ± 6.5%) than with the YOUPI software (p< 0.05).

Gating on the real cells, the rate of CD20+ B cells (**Figure 7B**), CD3+ T cells (**Figure 7B**), CD68+ macrophages (**Figure 7C**) and PanKeratin+ epithelial cells (**Figure 7D**) were not significantly different between YOUPI software and Ilastik and CellProfiler, and QuPath softwares. Only Ilastik and CellProfiler seems to detect fewer CD3+ T cells. The rates of CD20 and CD3 double-positive cells were also evaluated. Since CD20 and CD3 markers belong to B and T lymphocyte lineages, respectively (25), one single lymphocyte cannot express both markers. As shown in **Figure 7E**, no significant difference in the rates of false double-positive cells for Ilastik and CellProfiler, QuPath, and YOUPI software (3.9 ± 6.3% vs. 4.0 ± 5.4% vs. 4.2 ± 5.5%, p > 0.05). Similarly, the rates of PanKeratin and CD8 double-positive cells were evaluated. PanKeratin is an epithelial cell marker (26), while CD8 is a T-cell subset marker (25). Both cannot be co-expressed by a single cell. Again, the rate of false double PanKeratin and CD8 positive cells was low, and there were no differences among the three methods of segmentation (0.9 ± 1.7% vs. 0.9 ± 1.4% vs. 1.0 ± 1.1%, p > 0.05) (**Figure 7D**).

Consistently, correlation between QuPath vs YOUPI, Ilastik and CellProfiler vs YOUPI, and Ilastik and CellProfiler vs QuPath have been evaluated (**Supplementary Figure 3**). Strong correlation was identified for all analyses but Ilastik and CellProfiler vs YOUPI and Ilastik and CellProfiler vs QuPath for the percentage or real cells.

Performed on separated tissues, in which the number of total cells is different, the percentages of real cells and all cell subsets are significantly different only with Ilastik and CellProfiler compared to QuPath and YOUPI (**Supplementary Figure 4**).

## 4 Discussion

We introduced YOUPI, a cell segmentation tool for images generated by the Hyperion IMC intended for non-computer-friendly users. YOUPI works with U-Net. Three post-processing steps were added to obtain better control over the final segmentation mask. This mask provides access to single-cell data *via* CSV files for downstream supervised (manual gating strategy) or unsupervised (t-SNE, Phenograph, etc.) analysis whose performance falls between that of Ilastik and that of QuPath (**Supplementary Figure 5**).

The cell segmentation of tissue images can thus be performed using existing software, such as Ilastik and CellProfiler, or QuPath. Our experiences indicate that they require specific skills in image processing (e.g., image format conversion) and analysis to efficiently achieve cell segmentation. They rely on multiple third-party tools (ImageJ, Python scripts, etc.) and are thus barely accessible to non-bioinformatician users. Ease-of-use has guided our development of YOUPI. In contrast to the other tools, the all-in-one graphical interface can be used instinctually by non-computer scientists. In addition, two CSV files are generated. The first is a CSV file containing the mean intensity of markers, which scientists commonly use. However, aberrant high-intensity pixels are frequently identified in IMC images. The second is a CSV file containing the median intensity allowing to ignore these artifacts that can impact downstream analysis. It was added to the YOUPI software during the post-processing step to remove aberrant pixels.

Furthermore, among all existing tools or pipelines allowing for cell segmentation, the Ilastik and CellProfiler analysis of a given ROI requires approximately four hours of work, and QuPath software requires thirty minutes. With YOUPI, an inexperienced computer user obtains the segmentation mask in less than 10 minutes, including choosing markers of interest, thus making YOUPI a tool that is easy to use and delivers results quickly.

The segmentation results of the U-Net network are obtained with a set of essential steps. Manual segmentation is a crucial aspect of training to obtain reliable data. To obtain prebuilt binary images, we decided to use masks generated by Ilastik and CellProfiler softwares. Thus, QuPath was not useful since it does not consider membrane markers. Several weeks were required to manually correct the binary image patches. Although manual errors could have been made, the U-Net learned to perform segmentation as efficiently as Ilastik and CellProfiler, and as QuPath software at detecting the number of cells and of false-positive cells based on the corrected images. It proved to be the most efficient at detecting real cells. Correlation analyses of all tissues together, as well as, analyses on separated tissues, indicate that the Ilastik and CellProfiler detection method hardly match with QuPath and YOUPI software for the detection of real cells and some cell subsets. Segmentation results with YOUPI and QuPath are similar. The mean IoU value confirmed the robustness of the cell segmentation results obtained with YOUPI.

Although CD3, CD20, CD8, and PanKeratin molecules are expressed on distinct populations of cells (25, 26), some CD3 and CD20 double-positive cells or PanKeratin and CD8 double-positive cells could nevertheless be detected. It has recently been found that CD3+ T lymphocytes can express CD20 in blood and tissue (27) with potential relevance to human diseases (28), indicating the possible existence of a few real CD3+ and CD20+ double-positive cells. Alternatively, with high cellular density and depending on the thickness of the tissue section, touching or overlapping cells may be detected, given the possibility of detecting unexpected, rare PanKeratin and CD8 double-positive cells due to IMC technology’s limitations.

In conclusion, the image patches segmented to train YOUPI software came from different tissues (salivary gland, intestine, small cell lung cancer, and non-small cell lung cancer) to ensure the heterogeneity of cell types and shapes to train the U-Net. U-Net is a network specifically developed to analyze biomedical images, which allows for good performance from a restricted annotated dataset (18). Nonetheless, segmentation coming from a neural network will never be flawless. However, with the inclusion of additional post-processing steps (**Figure 5**), obvious mistakes the neural network could make are controlled; thus, YOUPI is safe.

YOUPI’s overall performance is comparable with that of other segmentation tools, despite relying on different approaches with different flaws. The naive mathematical approaches of Ilastik and CellProfiler can lead to the identification of non-existent cells, while the empirical threshold approach of QuPath can result in oversized shapes. Different intensities of image colorization can impact the quality of segmentation masks generated by YOUPI’s U-Net. It would be useful to apply these algorithms together to overcome their respective flaws. Therefore, the user can build an image that will be segmented according to the markers in which he or she is interested.

## Data availability statement

The raw data supporting the conclusions of this article will be made available by the authors, without undue reservation.

## Ethics statement

The studies involving human participants were reviewed and approved by the Comité de Protection des Personnes Ouest VI, Brest, Boulevard Tanguy Prigent, 29200 Brest, France. Written informed consent for participation was not required for this study in accordance with the national legislation and the institutional requirements. 

## Author contributions

YS, PH, NF, and CJ designed the study, and YS, PH, and MR analyzed generated data. PH performed the image acquisition, and YS developed the YOUPI tool with the support of NF. YS, PH, NF and CJ wrote the manuscript, and J-OP participated in the text edition. All authors contributed to the article and approved the submitted version.

## Supplementary material

Supplementary Figure 1 Individual staining of the markers used for the cell segmentation. Salivary gland, lung, intestine and kidney tissues were stained and images acquired with the Hyperion. Membrane markers (green) and nuclei (red) selected for cell segmentation as described in are shown individually (upper images) and with DNA staining (lower images).

Supplementary Figure 2 Segmentation of cells from kidney tissue with YOUPI. Kidney tissue was prepared with the panel described in . After acquisition of image with the Hyperion, the process of cell segmentation was performed using the YOUPI software without preliminary training phase. Membrane markers (green) and nuclei (red) are superposed with the YOUPI-generated mask (gray). Examples of superposition are shown (white squares).

Supplementary Figure 3 Correlation of YOUPI performance with other methods. Coefficient of correlation for the number of total cells, the percentage of real cells, the percentage of CD20+ B cells, the percentage of CD3+ T cells, the percentage of CD68+ macrophages, the percentage of PanKeratin+ epithelial cells, and the percentage of double CD3+CD20+ cells, and the percentage of double PanKeratin+CD8+ cells are shown between QuPath vs YOUPI, Ilastik and CellProfiler vs YOUPI, and Ilastik and CellProfiler vs QuPath.

Supplementary Figure 4 Cell segmentation performance of YOUPI on separate tissues. Four ROI from salivary glands, six ROI from lung and eight ROI from intestine were segmented with Ilastik and CellProfiler, QuPath and YOUPI software. The number of total cells, the percentage of real cells, the percentage of CD20+ B cells, the percentage of CD3+ T cells, the percentage of CD68+ macrophages, the percentage of PanKeratin+ epithelial cells, and the percentage of double CD3+CD20+ cells, and the percentage of double PanKeratin+CD8+ cells were calculated for each tissue and compared. *p< 0.05, **p< 0.01, ns, non-significant.

Supplementary Figure 5 Unsupervised downstream analysis following cell segmentation. Four ROI from salivary glands were segmented with Ilastik and CellProfiler, QuPath and YOUPI software. **(A)** The CSV files generated by the three approaches were merged for dimension reduction analysis with tSNE. **(B)** Clustering was performed with phenograph analysis, and the abundance of the clusters with each software and the phenotype of the cell clusters were determined.
