# Supplementary figures and images for: YOUPI: Your powerful and intelligent tool for segmenting cells from imaging mass cytometry data
Source: Front Immunol. 2023 Mar 2;14:1072118. doi: 10.3389/fimmu.2023.1072118 (PMC10019895; doi:10.3389/fimmu.2023.1072118)

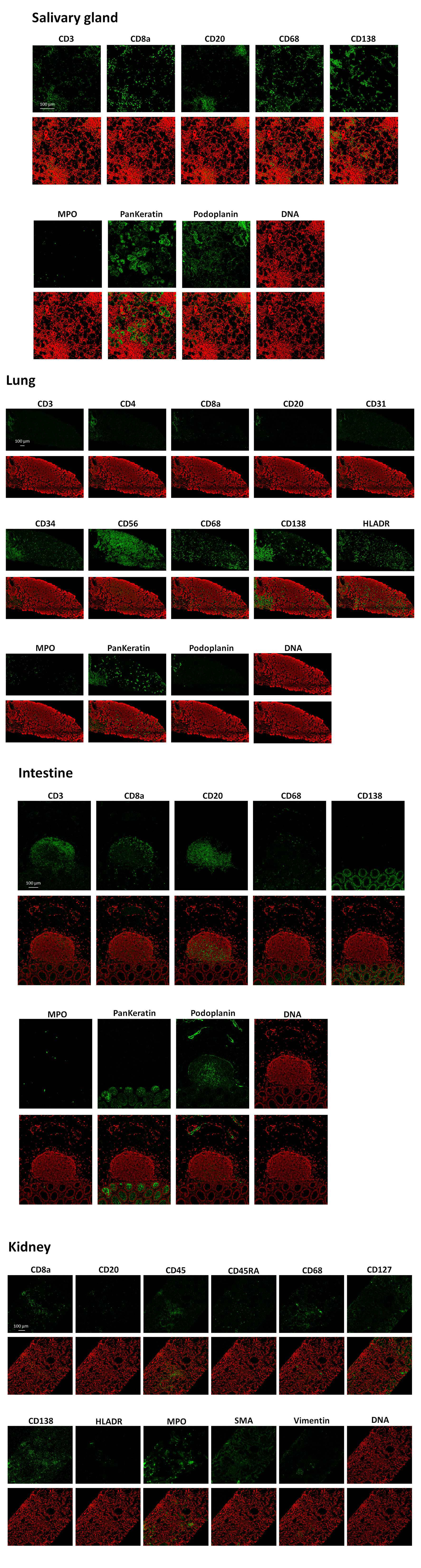

Supplement: Supplementary Figure 1 — Individual staining of the markers used for the cell segmentation. Salivary gland, lung, intestine and kidney tissues were stained and images acquired with the Hyperion. Membrane markers (green) and nuclei (red) selected for cell segmentation as described in are shown individually (upper images) and with DNA staining (lower images). [file Image_1.jpeg]

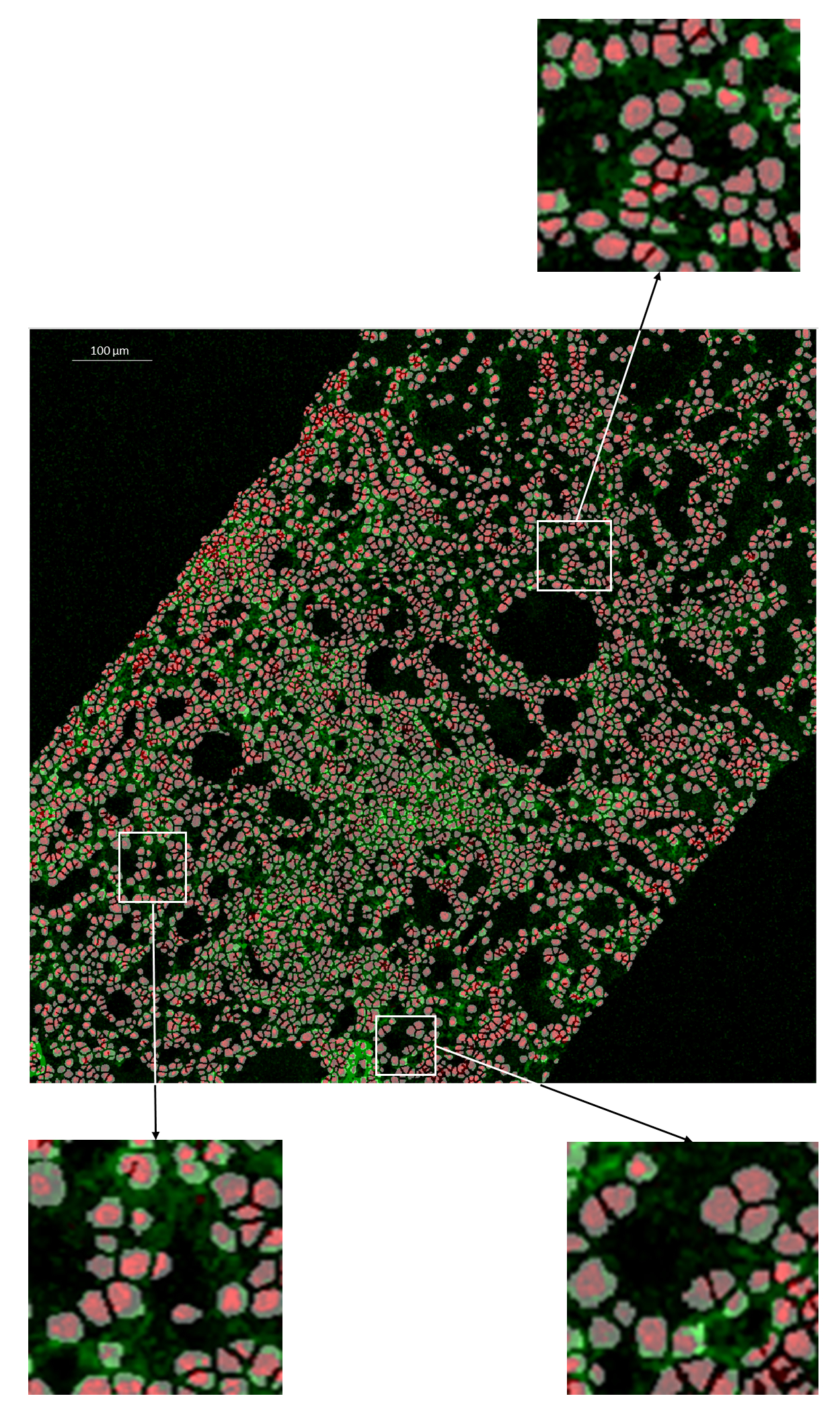

Supplement: Supplementary Figure 2 — Segmentation of cells from kidney tissue with YOUPI. Kidney tissue was prepared with the panel described in . After acquisition of image with the Hyperion, the process of cell segmentation was performed using the YOUPI software without preliminary training phase. Membrane markers (green) and nuclei (red) are superposed with the YOUPI-generated mask (gray). Examples of superposition are shown (white squares). [file Image_2.jpeg]

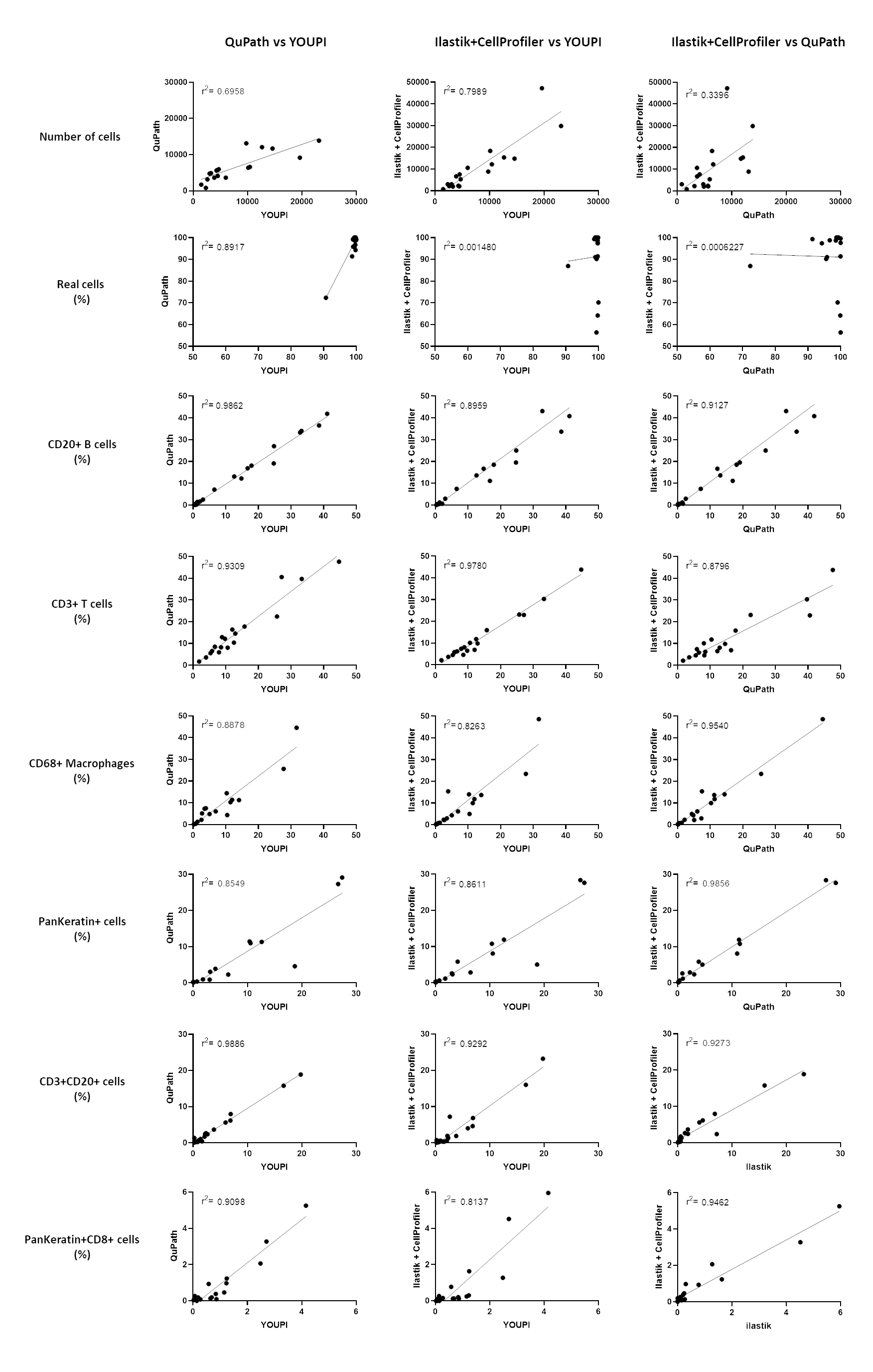

Supplement: Supplementary Figure 3 — Correlation of YOUPI performance with other methods. Coefficient of correlation for the number of total cells, the percentage of real cells, the percentage of CD20+ B cells, the percentage of CD3+ T cells, the percentage of CD68+ macrophages, the percentage of PanKeratin+ epithelial cells, and the percentage of double CD3+CD20+ cells, and the percentage of double PanKeratin+CD8+ cells are shown between QuPath vs YOUPI, Ilastik and CellProfiler vs YOUPI, and Ilastik and CellProfiler vs QuPath. [file Image_3.jpeg]

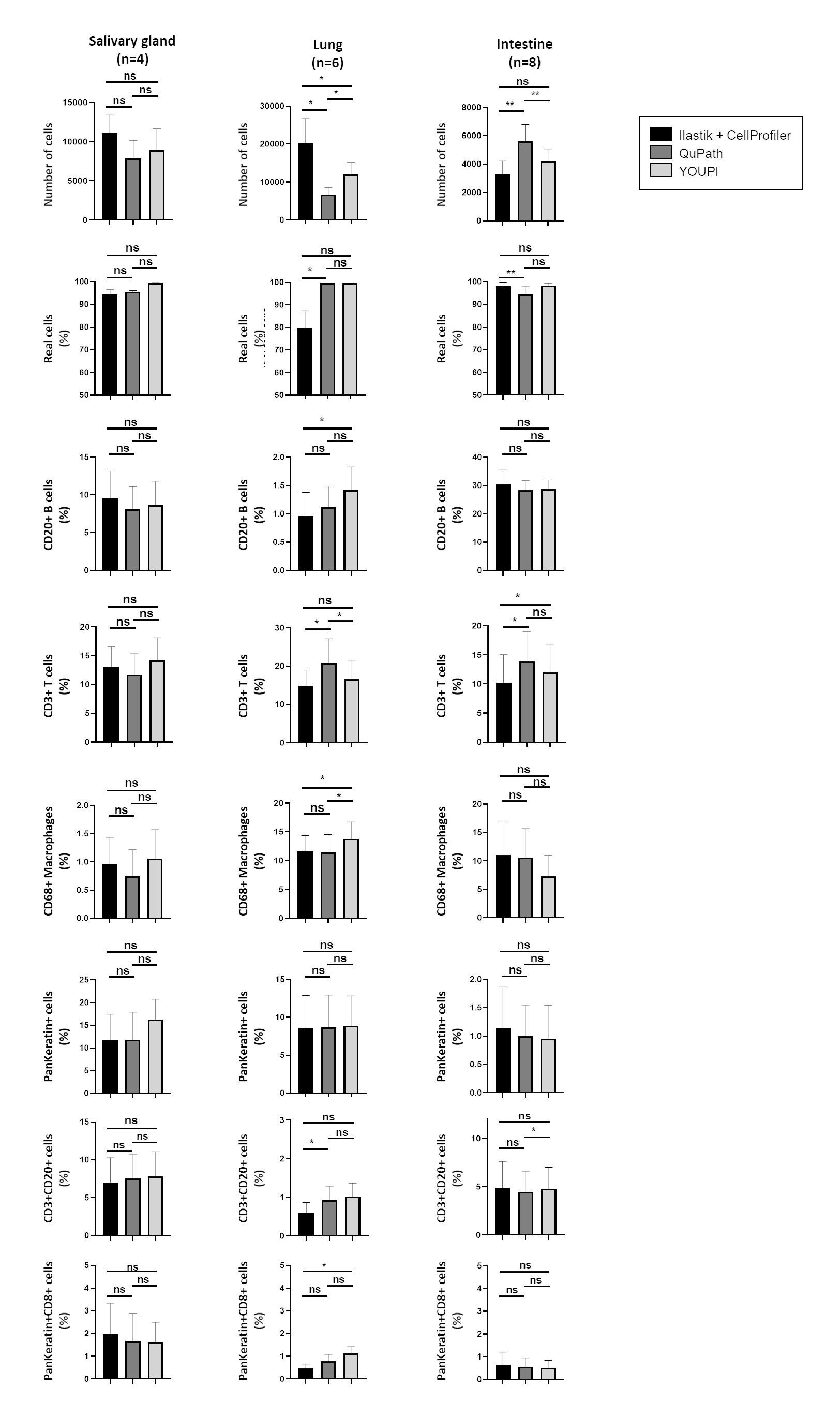

Supplement: Supplementary Figure 4 — Cell segmentation performance of YOUPI on separate tissues. Four ROI from salivary glands, six ROI from lung and eight ROI from intestine were segmented with Ilastik and CellProfiler, QuPath and YOUPI software. The number of total cells, the percentage of real cells, the percentage of CD20+ B cells, the percentage of CD3+ T cells, the percentage of CD68+ macrophages, the percentage of PanKeratin+ epithelial cells, and the percentage of double CD3+CD20+ cells, and the percentage of double PanKeratin+CD8+ cells were calculated for each tissue and compared. *p< 0.05, **p< 0.01, ns, non-significant. [file Image_4.jpeg]

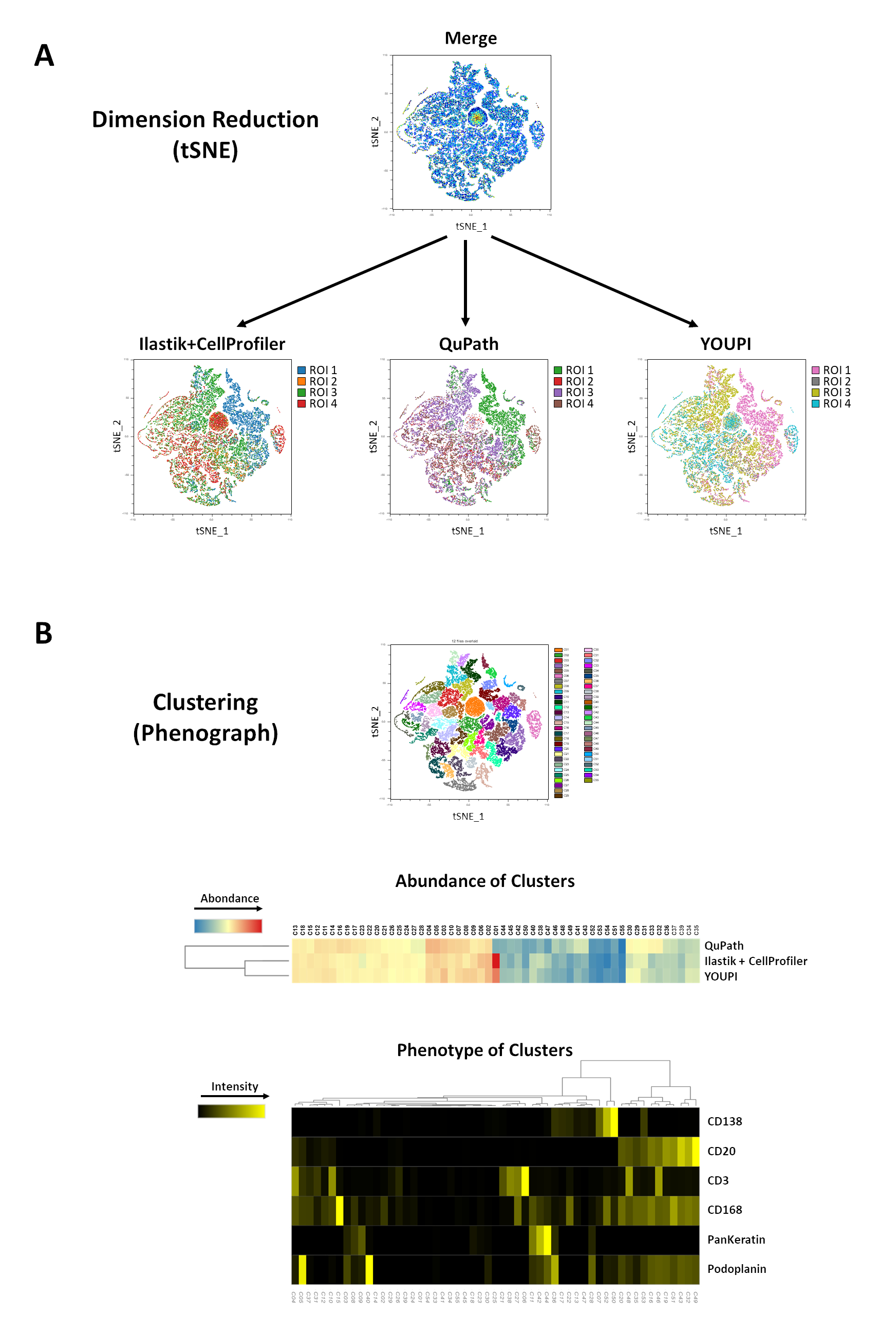

Supplement: Supplementary Figure 5 — Unsupervised downstream analysis following cell segmentation. Four ROI from salivary glands were segmented with Ilastik and CellProfiler, QuPath and YOUPI software. (A) The CSV files generated by the three approaches were merged for dimension reduction analysis with tSNE. (B) Clustering was performed with phenograph analysis, and the abundance of the clusters with each software and the phenotype of the cell clusters were determined. [file Image_5.jpeg]
